# Role of FDG-PET/CT in Assessing the Correlation Between Blood Pressure and Myocardial Metabolic Uptake 

**DOI:** 10.22038/aojnmb.2019.41530.1282

**Published:** 2020

**Authors:** Chaitanya Rojulpote, Siavash Mehdizadeh Seraj, Mahdi Zirakchian Zadeh, Divya Yadav, William Y. Raynor, Esha Kothekar, Abdullah Al-Zaghal, Thomas J. Werner, Oke Gerke, Poul Flemming Høilund-Carlsen, Abass Alavi

**Affiliations:** 1 Department of Radiology, Perelman School of Medicine, University of Pennsylvania, Philadelphia, United States; 2 Department of Radiology, Children’s Hospital of Philadelphia, Philadelphia, United States; 3 Department of Nuclear Medicine, Odense University Hospital, Odense, Denmark; * These authors shared last authorship

**Keywords:** FDG, PET/CT, Myocardial metabolic uptake, Blood pressure

## Abstract

**Objective(s):**

We aimed to assess the association between blood pressure and LV myocardial uptake of FDG, hypothesizing that subjects with raised blood pressure would have higher FDG uptake.

**Methods:**

We analyzed 86 healthy controls who underwent PET/CT imaging 180 minutes following FDG (4 MBq/Kg) administration. LV myocardial analysis was performed on axial sections using standard operator guided computer software (OsiriX MD). The average LV myocardial SUV_mean_ (MSUV_mean_) was calculated for each subject. Subjects were assessed according to the 2017 ACC/AHA guidelines for high blood pressure in adults. Mean arterial blood pressure (MABP) was calculated for each patient. Regression models were employed for statistical analysis.

**Results:**

The association of MSUV_mean_ was more pronounced with DP (r=0.32, p=0.003) than SP (r=0.28, p=0.010); MABP was comparable (r=0.33, p=0.002). Correlations of MSUV_mean_ with categorized BPs were: normal SP (r=0.27, p=0.010), elevated SP (r=0.28, p=0.009), stage 1 SP (r=0.27, p=0.010), stage 2 SP (r=0.28, p=0.008); normal DP (r=0.33, p=0.001), stage 1 DP (r=0.34, p=0.001), stage 2 DP (r=0.35, p=0.001). Multivariate analysis demonstrated DP (p=0.006), MABP (p=0.007), and SP (0.026).

**Conclusion:**

LV myocardial FDG uptake was higher in subjects with elevated blood pressure and correlated positively with SBP and in particular DBP and MABP.

## Introduction

 According to the National Institute of Health’s heart disease and stroke statistics 2017 update, cardiovascular disease (CVD) was the most common underlying cause of death in the world in 2013, accounting for 30.3%-32.9% of all global deaths (1). It is well known that hypertension is an important risk factor for the CVD, with higher blood pressures correlating to higher risks of 

mortality (2). The total number of adults with hypertension is predicted to increase to a total of approximately 1.56 billion by 2025, compared to 972 million of the global adult population in the year 2000 (3). 

 High BP can cause left ventricular myocardium to undergo certain adaptive changes as a compensatory mechanism in response to the high systemic pressures. This structural adaptation can lead to LV hypertrophy which subsequently gives rise to a number of cardiovascular disorders such as coronary artery disease (CAD), ischemic heart disease (IHD) and ultimately heart failure (HF) (4). The baseline pressure is known as diastolic pressure and is the residual pressure exerted on the aorta as the left ventricle relaxes between beats. The pressure that is seen when the heart pumps blood is known as systolic pressure and is the force that blood exerts on the arterial walls when the left ventricle contracts to pump blood to the peripheral organs and tissues. Mean arterial pressure incorporates systolic and diastolic pressures as one. It is calculated by taking the sum of two-thirds of diastolic pressure and one-third of systolic pressure. Mean arterial pressure is useful as it can help to understand overall blood flow. Elevation of these pressures is implicated in the causation of cardiovascular disease. 

 The clinical applications of fluorine-18-fluorodeoxyglycose (^18^F-FDG) positron emission tomography/computer tomography (PET/CT) are becoming increasingly popular in identifying non-oncologic conditions (5-7). In particular, the use of ^18^F-FDG PET/CT in cardiovascular has proved to be effective in identifying underlying myocardial changes at a molecular level as opposed to other conventional imaging modalities such as stress echocardiography, cardiac magnetic resonance, and transesophageal studies. Fluorine-18-FDG is analogous to glucose and will be rapidly taken up by the hypertrophied heart. This is very similar to cancer metabolism where increased ^18^F-FDG uptake is a sign of cell growth and proliferation (8). The combination of PET/CT molecular imaging with ^18^F-FDG substantially increases the diagnostic accuracy in detecting structural cardiovascular changes at a molecular level in subjects with high BP. It may also provide an insight into disease progression and subsequent development of complications. There is limited evidence to accurately quantitate myocardial uptake and so we aim to provide a novel method by means of trans-axial segmentation to quantify myocardial FDG uptake in relation to BP. 

 To our knowledge, there has not been another study that examined the correlation between ^18^F-FDG uptake in the LV with BP. The aim of this study is, therefore, to assess the role of ^18^F-FDG PET/CT imaging in assessing LV glucose metabolism in relation to BP. Our hypothesis is that subjects with raised BP would have higher uptake of ^18^F-FDG in the LV.

## Methods

*Subjects*

 We examined a total of 86 subjects from the prospective study known as “Cardiovascular Molecular Calcification Assessed by ^18^F-FDG PET/CT (CAMONA)” in Odense, Denmark. The CAMONA study was approved by the Danish National Committee on Biomedical Research Ethics as well as registered at ClinicalTrials.gov (NCT01724749). The study was undertaken in concordance with the Declaration of Helsinki and all subjects provided written informed consent. Subjects in this population were checked for the presence of malignancy, immunodeficiency syndrome, autoimmune disease, pregnancy, sarcoidosis, amyloidosis, endocarditis, symptoms suggestive of CVD such as syncope, chest pain, and shortness of breath, as well as prescription medications were considered as the exclusion criteria. 

 As we planned to determine the role of ^18^F-FDG uptake on PET/CT, we excluded subjects who did not have both the standardized PET scan and attenuated CT scan. We excluded 1 subject due to an inability of the Osirix MD platform to allow for correction of weight. All patients suffered from high blood pressure in their medical history and none of the patients took blood pressure lowering medications. Standard uptake value is defined from the following equation: radioactive concentration in tissue/ (injected dose/subject body weight). And thus, if a subject’s weight could not be considered, the subject was excluded. After these exclusions were made, we had a total of 86 subjects with a mean age of 46 years, ranging from 21-66 years ±13.5 SD (Table 1).

 Subjects’ BP was recorded as a baseline characteristic prior to PET scans performed. After 5 minutes of rest in a quiet room and in a supine position, BP was recorded three times by an automatic device. The last two values were averaged for the study. The 2017 guidelines for high BP in adults as set forth by the American College of Cardiology/American Heart Association Task Force on Clinical Practice Guidelines was followed (9). This guideline was preferred over the European Guidelines because it establishes a lower threshold for identifying and targeting individuals with high blood pressure. MABP was calculated by taking the sum of two-thirds of DP and one-third of SP.

**Table 1:** Subject demographic

**Total (N=86)**	
**Age**	46±13.5 (32.5-59.5)
**Systolic blood pressure (mm Hg)**	127.2±15.3 (111.9-142.5)
**Diastolic blood pressure (mm Hg)**	76.5±10.0 (66.5-86.5)
**Low density lipoprotein (mmol/L)**	3.0±0.8 (2.2-3.8)
**Total cholesterol (mmol/L)**	4.9±0.9 (4.0-5.8)
**Triglycerides (mmol/L)**	1.1±0.7 (0.4-1.8)
**Homocysteine (umol/L0**	8.8±2.5 (6.3-11.3)
**Plasma glucose (mmol/L)**	5.5±0.5 (5.0-6.0)
**HbA1c (mmol/mol)**	33.8±4.1 (29.7-37.9)
**Smokers ** Active Ever Never	3 31 52

Values are mean ±SD, n (%). HbA1c = Glycated hemoglobin.

*Quantitative image analysis*

 All subjects underwent ^18^F-FDG PET/CT imaging with an established and uniform protocol (GE Discovery STE, VCT, RX, and 690/710). Subjects were made to observe an overnight fast of 8 hours and a blood glucose measurement ensuring a concentration below 8mmol/L. Fluorine-18- FDG PET/CT imaging was performed at approximately 45, 90 and 180 minutes following the intravenous injection of 4 MBq/kg of ^18^F-FDG of body weight. In our study, we used only 180-minute scans as all the subjects had at least a scan done at 180 minutes as some subjects did not undergo 45- and 90-minute scans. 

 During this time, subjects were asked to remain in a quiet room so as to minimize pretesting anxiety and maintain a normal heart rate as tachycardia and BP changes could affect metabolism. These images were produced using one of several PET/CT systems (GE Discovery STE, VCT, RX, and 690/710, USA). Positron emission tomography images were corrected for attenuation, scatter, scanner dead time, and random coincidences. Low-dose CT imaging (140 kV, 30-110 mA, noise index 25, 0.8 seconds per rotation, slice thickness 3.75 mm) was performed for attenuation correction and anatomic referencing with PET images. 

**Figure 1:**
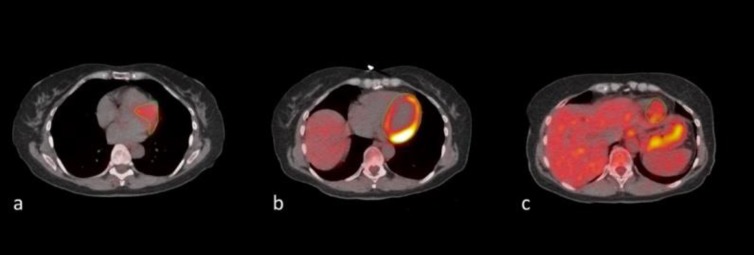
These scans belong to a 62 years old female. Regions of interest were manually drawn around the anatomical borders of the left ventricle on fused ^18^F-FDG PET/CT axial slices. (a=superior, b=middle, c= inferior)

 Semi-quantification of LV ^18^F-FDG uptake was performed by a trained physician by manually placing a free-hand region of interest (ROI) around the LV on every slice of the axially oriented PET/CT images using a DICOM viewer (Osirix MD Software; Pixmeo SARL, Bernex, Switzerland) (Figure 1). 

We meticulously excluded ^18^F-FDG activity arising from the aortic wall and cardiac valves by eliminating these areas from the ROI. For every ROI, the standard ^18^F-FDG activity was determined. After generating the standard uptake values from the ROI, we calculated the mean standard uptake value (SUV) for each subject. To do this, we used the SUV of each PET scan and multiplied with the area of each scan. Then we took the sum of these SUV and divided it by the sum of the area of each scan to finally derive the SUV_mean_. 

*Statistical analysis*

 The association between the severity level of the BP and the SUV_mean_ of the LV was evaluated. The Pearson correlation coefficient for the mean ^18^F-FDG uptake of the LV region and BP was performed for SP, DP and MABP. Unpaired t-test and ANOVA was done to assess the differences in SUV_mean_ of normal BP with high SP and DP. Regression plots and box plots were generated to help with visualization of data. Multivariable linear modelling was applied for each BP measurement (i.e. SP, DP, MABP) using available demographic and baseline information (age, gender, LDL cholesterol, total cholesterol, homocysteine, HbA1c, and fasting plasma glucose). Variance inflation factors were investigated in order to prevent inclusion of information redundant variables (10); to this end, LDL cholesterol was chosen and total cholesterol was omitted. R-squared and adjusted R-squared values were reported as measures for model fit. A P value <0.05 was taken as significant. Bland-Altman Limits of Agreement (BA LoA) were used to visually display intra- and inter-rater agreement of SUV_mean_ ((11-13). We used statistical software package (SPSS Version 25.0), STATA/MP 15 (StataCorp, College Station, Texas 77845 USA) and Microsoft Excel (2016 Version 16.16.2) for the statistical analysis and generating Figures. 

## Results

**Figure 2:**
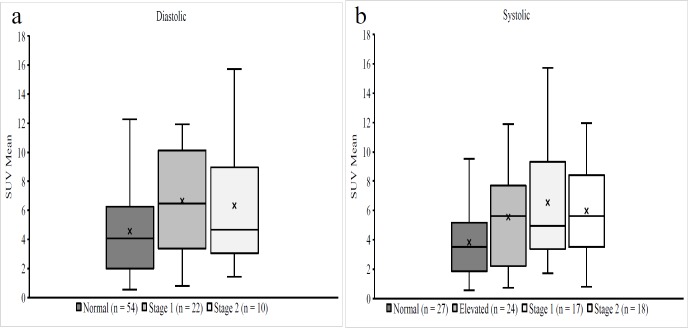
Box plot comparing SUV_mean_ of systolic and diastolic blood pressure

 In this study, we included 86 subjects. The average SUV_mean_ in the LV was found to be 5.3±3.4. The average SP and DP was found to be 127.2±15.3 and 76.5±10, respectively. (Figure 2).

 Subjects with a normal SP had an average SUV_mean_ of 3.8±2.4 and normal DP had an average SUV_mean_ of 4.5±2.9. Subjects with an elevated, stage 1 and stage 2 systolic hypertension had an average SUV_mean_ of 5.5±3.3, 6.5±3.9 and 5.9±3.5, respectively. Subjects with stage 1 and stage 2 diastolic hypertension had an average SUV_mean_ of 6.6±3.4 and 6.3±4.5, respectively. (Figure 2).

**Figure 3:**
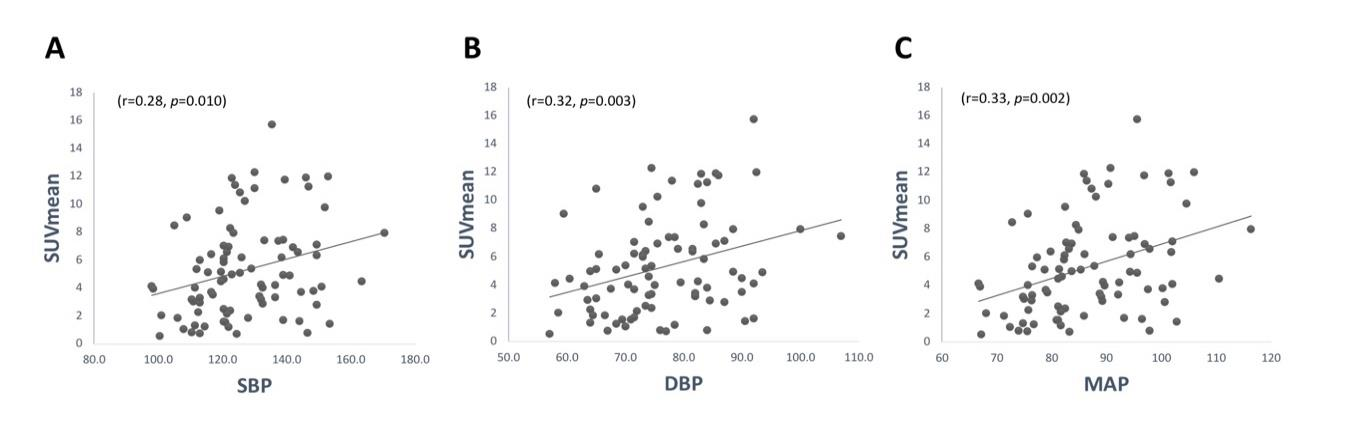
Correlations between SUV_mean_ and Systolic, Diastolic, and Mean Arterial Pressure. Significant correlations were present for ^18^F-FDG uptake in all three pressures; the standard error for the slope estimate was 0.023, 0.035, and 0.032 in A, B, and C, respectively

**Figure 4:**
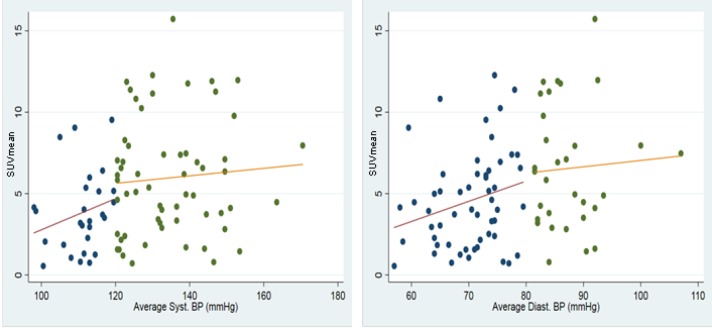
Visual depiction of correlations between normal SP (<120mmHg) and SUV_mean_ (red) vs. high SP (>120mmHg) and SUV_mean_ (orange) (left); Normal DP (<80mmHg) and SUV_mean_ (red) vs. high DP (>80mmHg) and SUV_mean_ (orange) (right)

 The linear correlation coefficient between SUV_mean_ and DP, SP and MABP was found to be significant (r=0.32, P=0.003) (r=0.28, P=0.010) and (r=0.33, P=0.002), respectively. Subjects were then categorized according to the American College of Cardiology/American Heart Association Task Force on Clinical Practice Guidelines for High Blood Pressure in Adults and the results are as follows: normal SP (n=27, 18 females, 9 males, age 42.1), elevated SP (n=24, 7 females, 17 males, age 45.8), stage 1 SP (n=17, 6 females, 11 males, age 48.9), stage 2 SP (n=18, 9 females, 9 males, age 49.2), normal DP (n=54, 27 females, 27 males, age 44.5), stage 1 DP (n=22, 8 females, 14 males, age 50.0), and stage 2 DP (n=10, 5 females, 5 males, age 44.9). Linear correlations of SUV_mean_ and SP were as follows: normal (r=0.27, P=0.010), elevated (r=0.28, P=0.009), stage 1 (r=0.27, P=0.010), stage 2 (r=0.28, P=0.008) (Figure 3). Linear correlations of SUV_mean_ and DP were as follows: normal (r=0.33, P=0.001), stage 1 (r=0.34, P=0.001), stage 2 (r=0.35, P=0.001) (Figure 3). The standard error of the estimate of the line of regression for graphs A, B and C were 0.023, 0.035, and 0.032. These standard errors of the estimates were added to the legend of Figure 3. Linear correlation graphs were generated to display the comparison between normal and high SP as well as normal and high DP (Figure 4). Linear correlation of SUV_mean_ and heart rate was found to be insignificant (r=0.03, P=0.817). Variable selection by means of univariate analysis was not performed as a variable may be non-significant in univariate, but significant in multivariate analysis (14).

 Multivariate linear regression analysis displayed significance with DP (P=0.006) (Table 2), SP (P=0.026) (Table 3), and MABP (P=0.007) (Table 4). ANOVA analysis of SUV_means_ of SP and DP was significant (P=0.04 and 0.03, respectively) (Table 5). 

**Table 2:** Multivariable linear regression of DP (N=78, R-squared=0.16, adjusted R-squared=0.07)

Variable	Point Estimate	95% CI	P-value
Average DP	0.12	0.03 – 0.20	0.006
Gender	0.57	-1.04 – 2.18	0.48
Age	0.35	-0.02 – 0.09	0.25
LDL cholesterol	0.49	-0.49 – 1.47	0.32
Fasting plasma glucose	-0.76	-2.48 – 0.95	0.38
HbA1c	-0.09	-0.29 – 0.11	0.38
Homocystine	-0.02	-0.35 – 0.30	0.89

**Table 3:** Multivariable linear regression of SP (N=78, R-squared=0.13, adjusted R-squared=0.04)

Variable	Point Estimate	95% CI	P-value
**Average SP**	0.06	0.01 – 0.12	0.026
**Gender**	0.42	-1.22 – 2.07	0.61
**Age**	0.03	-0.03 – 0.09	0.34
**LDL cholesterol**	0.64	-0.34 – 1.62	0.19
**Fasting plasma glucose**	-0.39	-2.12 – 1.33	0.65
**HbA1c**	-0.09	-0.29 – 0.11	0.37
**Homocystine**	0.29	-0.29 – 0.35	0.86

**Table 4:** Multivariable linear regression of MABP (N=78, R-squared=0.16, adjusted R- squared=0.07)

**Variable**	**Point Estimate**	**95% CI**	**P-value**
**Average MABP**	0.11	0.03 – 0.18	0.007
**Gender**	0.50	-1.11 – 2.11	0.54
**Age**	0.03	-0.03 – 0.09	0.31
**LDL cholesterol**	0.53	-0.44 – 1.50	0.28
**Fasting plasma glucose**	-0.62	-2.31 – 1.08	0.47
**HbA1c**	-0.10	-0.30 – 0.10	0.34
**Homocystine**	-0.01	-0.34 – 0.31	0.93

**Table 5:** Summary statistics for one-way ANOVA and pairwise comparisons by systolic and diastolic BP

**Predictors**	**SUV**_mean_	
	** F (df) **	**Mean (SD)**
**Systolic**	0.04* (3, 82)	
Normal: <120		3.82 (2.48)1
Elevated: 120-129		5.55 (3.38)2
Stage 1: 130-139		6.54 (3.99)3
Stage 2: >140		5.97 (3.54)4
**Diastolic**	0.03* (2, 83)	
Normal: <80		4.55 (2.97)a
Stage 1: 80-89		6.64 (3.46)b
Stage 2: >90		6.31 (4.57)c

Note. ^*^p < .05. Systolic groups are labelled with numerical superscripts and significance was found between the following groups: 1 & 2, 1 & 3, 1 & 4. Diastolic groups are labelled with alphabetical superscripts and significance was found between the following groups: a & b.

T-test comparisons were done to assess the SUV_mean_ of the varying SP groups and are as follows: normal and elevated systolic (P=0.04), normal and stage 1 systolic (P=0.01), and normal and stage 2 systolic (P=0.03). T-test comparisons for the diastolic groups are as follows: normal diastolic and stage 1 diastolic (P=0.01) and normal and stage 2 diastolic (P=0.2). The lack of statistical significance between normal diastolic and stage 2 diastolic may be due to the small sample of subjects in the stage 2 group.

*Repeatability studies*

**Figure 5:**
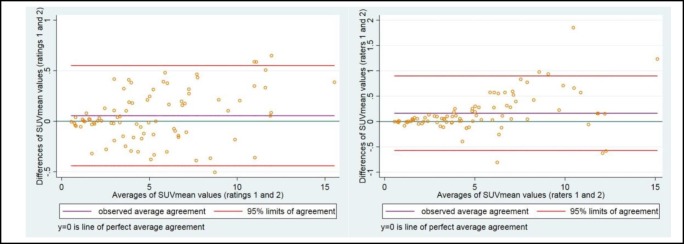
Bland-Altman plots displaying intra- (LEFT) and inter-rater agreement (RIGHT) of repeated SUV_mean_ measurements

 All 86 subjects in the study were selected for intra- and inter-rater repeatability testing. After completion of the LV myocardial image analysis, all scans belonging to these individuals were analyzed independently by another trained observer. This provided inter-rater repeatability for MSUV_mean_ values. To assess intra-rater variation, the initial observer repeated the analysis 4 weeks later to reduce recall bias. The reproducibility studies displayed excellent intra- and inter-rater repeatability for MSUV_mean_ values. Bland-Altman Limits of Agreement (BA LoA) were used to visually display intra- and inter-rater agreement of SUV_means_. (Figure 5) (Table 6).

**Table 6:** Bland-Altman limits of agreement

**Comparison**	**Estimated mean difference** **(Standard deviation)**	**Limits of agreement**
Rater 1: rating 1 vs. rating 2	0.055 (0.253)	-0.44 to 0.55
Rater 1 vs. rater 2	0.164 (0.376)	-0.57 to 0.90

## Discussion

 The increased ^18^F-FDG uptake on PET/CT as shown in this study provides evidence in support of the hypothesis that LV uptake is higher in subjects with higher BP as compared to subjects with normal BP. The difference in the average SUV_mean_ uptake in elevated (5.5), stage 1 (6.5) and stage 2 (5.9) systolic hypertension versus normal systolic (3.8) was found to be 1.7, 2.7, and 2.1, respectively. The difference in the average SUV_mean_ uptake in stage 1 (6.6) and stage 2 (6.3) diastolic hypertension versus normal diastolic (4.5) was found to be 2.1 and 1.8, respectively.

 In this study, we found that ^18^F-FDG uptake in the LV is positively correlated with SP, DP, and MABP in the univariate analysis. A multivariate analysis revealed significance with DP, SP, and MABP. Moreover, the LV ^18^F-FDG uptake was higher with higher BP levels in comparison with subjects with normal BP. When assessing the SUV_mean_ between normal SP group versus elevated SP group, stage 1 and stage 2 systolic hypertension, there was a clear significance in the uptake. Assessment of SUV_mean_ between normal DP versus stage 1 diastolic group displayed a significant difference in the uptake. However, we did not find a significant difference in ^18^F-FDG uptake between the SUV_mean_ of stage 2 DP group and normal subjects. We believe that this finding in the stage 2 diastolic group in comparison with the normal group is due to the small sample size of subjects with stage 2 diastolic hypertension (n=10). It may also be due to the effect of chronic hypertension on the heart, potentially resulting in myocardial fibrosis and a lower uptake. 

 The association of increased ^18^F-FDG uptake in relation to higher BP can be explained by an understanding of the compensatory adaptations of the heart. When the heart is faced with increased systemic vascular pressures, it will hypertrophy to overcome these pressures. These myocardial changes are seen in the myocytes and the interstitial compartment of the heart. The interstitial compartment is comprised mainly of fibroblasts and collagen. The increase in systemic vascular resistance causes the myocytes to hypertrophy. However, it is the excessive proliferation of fibrous tissue in the interstitial compartment in relation to the myocytes that facilitates this remodeling (15). The remodeling of the heart causes the LV to thicken, which hinders the ventricle’s ability to adequately pump blood to the body. The thickened LV results in a narrowing of the chamber and subsequent diastolic dysfunction. This forces the heart to work harder in order to pump blood to the rest of the body. An imbalance is created by an inadequate supply of oxygenated blood by the heart and the increased demand of oxygen from the body (16). It is important to note that although the LV progressively hypertrophies initially, it is followed by a reduced rate in growth due to myocyte apoptosis and fibrosis, leading to myocardial dysfunction.

 The hypertrophied LV, combined with an increased workload, compels at first the heart to utilize higher amounts of glucose (17-19). This is a shift from the healthy state in which the heart acquires the majority of its energy from the oxidation of fatty acids. Under normal conditions, the heart utilizes non-carbohydrate substrates such as fatty acids as the major metabolic pathway because it provides the highest energy yield per molecule of substrate that is metabolized. Free fatty acids such as palmitate provide the highest amount of ATP per molecule of substrate through the process of β-oxidation. In comparison, glucose provides significantly less ATP. However, glucose metabolism is more efficient in generating ATP for every oxygen molecule consumed as opposed to free fatty acids (20). This change in metabolism to glucose results in the loss of metabolic adjustability which is associated with increased reliance on glucose utilization and in turn contributes to the development of cardiac dysfunction (18, 21). Fatty acids are still utilized with this transition, though to a lesser degree, and glucose in higher amounts as compared to normal conditions. The switch to increased utilization of glucose is seen in subjects with LV hypertrophy in the setting of high BP, and the structural change that follows can be identified with appropriate diagnostic imaging, mainly ^18^F-FDG uptake as detected by PET/CT. 

 Our study has found that MABP was found to be closely related to ^18^F-FDG uptake (r=0.33). As MABP is a composite of both BP and DP, dichotomizing the pressure into its individual pressures reveals that DP is more pronounced (r=0.32) than SP (r=0.28). It has been shown that the SP correlates closely with LV mass. However, it is the DP that is more closely associated to LV wall thickness which reflects pure pressure load (15). Between the effects of these two pressures, there is a resultant hypertrophic effect on the LV. This hypertrophic change has been shown to increase the risk of developing CAD. The Framingham Study has shown that the occurrence of LV hypertrophy alone exceeds the overall risk of mortality than that which follows myocardial infarction (22). In fact, LV hypertrophy has been shown to be the most common myocardial structural abnormality associated with heart failure with preserved ejection fraction (23). The occurrence of myocardial fibrosis in LV hypertrophy is an important mechanism for the development of diastolic heart failure (24). The myocardial fibrosis occurs due to molecular mechanisms that activate the renin-angiotensin-aldosterone system which in turn promotes TGF-β expression and eventually heart failure (25).

 Our study has shown a clear correlation of ^18^F-FDG uptake of the LV in a group of subjects with increasingly higher BP as seen on PET/CT. This finding may have prognostic value in identifying individuals who may develop CVD in the future. This data is only made possible due to the capability of the PET scan, which provides information of the disease process at a molecular level and is not seen with other imaging modalities such as cardiac magnetic resonance (CMR). Though echocardiography is routinely used in practice due to its low cost and wide accessibility, it is operator and subject dependent, which can result in a high inter-observer variability (26). As disease processes often begin with functional changes at the cellular level, PET may detect changes at their earlier stages based on spatial resolution. This may provide an advantage over CT or CMR because they cannot detect changes until diseases start to cause changes in the overall structure of the organ or tissue. 

 Some may argue the use of single photon emission tomography (SPET) over PET for its relatively cheaper cost. However, Bateman et al. (2006) studied the diagnostic accuracy of PET and SPET and found it to be higher than that of SPET (27). There is also mounting data to support the prognostic value of PET scan as confirmed by others (28). 

 A limitation of this study was that we could not validate the ^18^F-FDG-PET/CT findings with histological data. This study aimed to characterize LV structural changes at a microscopic level and so it would have been advantageous to confirm if these presumed findings were actually present histologically. Another limitation of our study pertains to how BP was recorded in the subjects. As BP was recorded in a medical setting, it is possible to have the white coat hypertension effect on subjects as the presence of a doctor and being in a medical setting can cause a slight increase in BP. Furthermore, we were not able to ascertain the impact that duration of BP had on MSUV_mean_ as subjects with chronicity of BP elevation may have altered myocardial metabolism.

## Conclusion

 Fluorine-18-FDG uptake in the LV was found to increase with higher BP. Fluorine-18-FDG uptake was found to be more strongly correlated with DP and MABP than with SP. By semi-quantifying ^18^F-FDG uptake in the LV in hypertensive subjects, it may allow us to determine the future risk of CVD in subjects as well as take counter measures prior to the onset of clinical disease. 

## Sources of Funding

 The Jørgen and Gisela Thrane’s Philanthropic Research Foundation, Broager, Denmark, financially supported the CAMONA study. 

## Conflict of interest

 The authors declare that they have no conflicts of interest. 
